# Association Between Cutaneous Immune-Related Adverse Events and Efficacy of Immune Checkpoint Inhibitors in Advanced Non-Small Cell Lung Cancer

**DOI:** 10.3390/jcm14072499

**Published:** 2025-04-06

**Authors:** José Miguel Jurado, Vanesa Gutiérrez, Alexandra Cantero, Miguel-Ángel Berciano-Guerrero, Airam Padilla, Elisabet Pérez-Ruiz, Álvaro Montesa, Francisco Carabantes, Manuel Cobo

**Affiliations:** 1 Hospital Universitario Regional de Málaga, 29010 Málaga, Spain; vanesa_gutierrez78@hotmail.com (V.G.); alexandracantero.eecc@gmail.com (A.C.); migueberci@gmail.com (M.-Á.B.-G.); airampadillaalvarez@gmail.com (A.P.); eliperu@gmail.com (E.P.-R.); amontesa76@hotmail.com (Á.M.); fcarabantes@hotmail.com (F.C.); manuelcobodols@yahoo.es (M.C.); 2 Biomedical Research Institute of Malaga (IBIMA), 29010 Málaga, Spain

**Keywords:** cutaneous immune-related adverse events (cirAEs), immune checkpoint inhibitors (ICIs), non-small cell lung cancer (NSCLC)

## Abstract

**Background/Objectives**: Immune checkpoint inhibitors (ICIs) have transformed the treatment of patients with non-small cell lung cancer (NSCLC). Numerous studies have suggested that immune-related adverse events (irAEs) are associated with ICI efficacy and can affect any organ system. This study aims to evaluate the prognostic significance of cutaneous IrAEs (cirAEs) and their impact on the effectiveness of PD-1/PD-L1 inhibitors in real-world NSCLC data. **Methods**: We retrospectively collected NSCLC patients treated with ICI as first- or second-line therapy between 2015 and 2022 at a single institution. We evaluated the association between cirAEs and treatment efficacy, measured by objective response rate (ORR), progression-free survival (PFS), and overall survival (OS). Kaplan–Meier survival curves were generated, and log-rank tests were used for significance testing. Multivariable analysis was performed using Cox proportional hazards regression models. **Results**: A total of 510 patients were included in the analysis, with a median age of 62 years (range 34–85), and 75% of patients were males. CirAEs of any grade were observed in 139 patients (27.3%). Among patients assessed for efficacy, the ORR was significantly higher in those with cirAEs compared to those without (54.3% vs. 29.9%, *p* = 0.0001). At a median follow-up of 48 months, PFS (14.6 vs. 4.7 months, *p* = 0.0001) and OS (29 vs. 9.2 months, *p* = 0.0001) were significantly improved in patients with cirAEs. Patients with grade 1–2 cirAEs showed even greater survival benefits (PFS: median 14.9 months, *p* = 0.003; OS: median 30 months, *p* = 0.001). Multivariable analysis confirmed that the development of any cirAE was independently associated with significantly improved OS (hazard ratio [HR] 0.60, 95% confidence interval [CI] 0.44–0.80, *p* = 0.0001). The presence of multisystem ≥ 2 SOC irAEs, including cirAE, was strongly correlated with the greatest benefit from ICIs HR:0.51 (95% CI 0.35–0.74), *p* = 0.001. **Conclusions**: This study supports that cirAEs could be used as a potential marker of ICI efficacy in NSCLC. The development of multisystem cirAEs may prognose the greatest benefit of treatment.

## 1. Introduction

NSCLC remains the leading cause of cancer-related mortality in Western countries [1]. Recent advances with immune checkpoint inhibitors (ICIs) have significantly improved the prognosis for patients with many malignancies, including NSCLC [2,3].

ICIs are monoclonal antibodies targeting immune checkpoint molecules such as cytotoxic T-lymphocyte antigen-4 (CTLA-4), programmed cell death protein-1 (PD-1), and programmed cell death ligand-1 (PD-L1), thereby unleashing the immune system to attack tumor cells through T-cell activation [4].

In the first-line setting for advanced NSCLC without actionable genomic alterations, several large phase III trials have demonstrated that combining ICIs with chemotherapy significantly improves overall survival (OS) compared to chemotherapy alone, regardless of PD-L1 expression levels. Patients with PD-L1 expression ≥ 50% are often treated with ICI monotherapy, with or without platinum-based chemotherapy [5,6,7,8].

PD-L1 expression, evaluated through immunohistochemistry (IHC), remains the most widely used biomarker for predicting response to PD-1/PD-L1 inhibitors in NSCLC [9]. Different thresholds of PD-L1 expression guide treatment decisions, depending on the specific inhibitor approved.

Treatment with ICIs is associated with a distinct spectrum of inflammatory toxicities known as immune-related adverse events (irAEs), which result from heightened T-cell activity against antigens shared by both tumors and healthy tissues [10,11,12].

Several studies have demonstrated an association between the development of irAEs and improved efficacy of ICIs across multiple cancers [13,14,15]. However, whether this association applies universally to all irAEs or is limited to specific system/organ classes (SOCs) remains unclear [15,16].

SOC irAEs occurred most frequently in the endocrine organs and skin [17]. Cutaneous immune-related adverse events (cirAEs) are mostly described as rash and pruritus and tend to be mild. Management of low-grade toxicity is supportive with topical corticosteroids and antihistamines. Grade ≥ 3 events require treatment interruption, oral corticosteroids, and dermatologic consultation [18]. CirAEs can manifest as isolated skin toxicities or as part of systemic disorders involving multiple SOCs. Commonly affected systems include the skin, gastrointestinal tract, endocrine glands, and liver. Less frequently, irAEs involve the lungs, nervous system, kidneys, hematological system, musculoskeletal system, heart, or eyes [17].

Given the potential severity of these side effects, early recognition and effective management are crucial to ensure the safe continuation of treatment. This retrospective study aims to evaluate the prognostic significance of cirAEs and the effectiveness of PD-1/PD-L1 inhibitors in real-world NSCLC patients with longer follow-up. Finally, we also investigated whether there is a similar association between cirAEs with multiple SOCs and outcomes.

## 2. Materials and Methods

This retrospective study was conducted on a European population of patients with advanced NSCLC treated with PD-1 or PD-L1 inhibitors at Hospital Universitario Regional de Málaga between 1 March 2015 and 30 March 2022.

Study Population: Eligible patients included adults (≥18 years) with histologically or cytologically confirmed advanced NSCLC who had received at least one cycle of PD-1 or PD-L1 inhibitors as first- or second-line therapy. Key inclusion criteria included an Eastern Cooperative Oncology Group (ECOG) performance status (PS) of ≤2, measurable disease per RECIST v1.1, and adequate organ function, defined as: Renal function: Creatinine clearance > 50 mL/min. Liver function: Total bilirubin < 1.5 times the upper normal limit. Hematologic function: Absolute neutrophil count > 1000/µL, platelet count > 100,000/µL, and hemoglobin > 9 g/dL. Patients with actionable genomic alterations, such as EGFR mutations or ALK rearrangements, were excluded.

Data Collection: Clinical data were extracted from electronic medical records and included the following demographics and baseline tumor characteristics: age, gender, smoking status, histology, Kras status, PD-L1 expression levels, number of prior therapies, treatment history, toxicity profiles, dose modifications, and reasons for treatment discontinuation.

Adverse Event Assessment: IrAEs, including cirAEs, were graded using the Common Terminology Criteria for Adverse Events (CTCAE) v5.0. [19]. The specific definitions and grading criteria for pruritus and maculopapular rash were as follows. Pruritus, graded from mild (G1, localized) to severe (G3, widespread with systemic therapy indicated). Skin lesions covering <10%, 10–30%, and >30% of the body surface area were classified as grades 1, 2, and 3, with moderate to severe symptoms. Life-threatening conditions were categorized as grade 4 events.

Efficacy outcomes included: Objective Response Rate (ORR): Proportion of patients achieving complete response (CR) or partial response (PR) per RECIST v1.1 criteria. Progression-Free Survival (PFS): Time from treatment initiation to disease progression or death. Overall Survival (OS): Time from treatment initiation to death from any cause. Clinical and radiological tumor assessments were conducted according to RECIST v1.1 guidelines [20]. Response rates, progression-free intervals, and survival outcomes were analyzed in relation to the presence of cirAEs and multisystem irAEs.

Statistical Analysis: Qualitative variables are expressed as absolute and relative frequencies. For the comparisons of proportions, Fisher’s exact tests were used. PFS and OS curves were generated by using the Kaplan–Meier method, survival analysis, and the differences were evaluated using the log-rank test. Associations between cirAEs and efficacy outcomes (PFS, OS) were analyzed using univariate and multivariate Cox proportional hazards regression models [21]. The multivariate analysis was adjusted for relevant covariates with a univariate *p*-value of less than 0.1 including performance status, PD-L1 expression, smoking history, and tumor response. A *p*-value of <0.05 was considered statistically significant. Statistical analyses were performed using SPSS software version 22.0 (SPSS Inc., Chicago, IL, USA) and NCSS Statistical Software version 12 (NCSS, LLC, Kaysville, UT, USA).

## 3. Results

### 3.1. Patient Baseline Characteristics

A total of 510 patients with advanced NSCLC were included in this study. The median age was 62 years (range: 34–85 years). The clinicopathological characteristics and treatment data are summarized in Table 1. Among the cohort, 76% of patients were male and 24% were female. Most patients had an ECOG 0–1 (96), and 44% were current smokers. Adenocarcinoma was the predominant histological subtype (61%), followed by squamous cell carcinoma (34%). Most patients received immunotherapy as first line (61%). Treatments administered included immune checkpoint inhibitors (ICIs) monotherapy in 63% of patients, and 37% receiving concurrent chemo-immunotherapy.

### 3.2. Cutaneous Immune-Related Adverse Events

Of the total cohort, cutaneous irAEs (cirAEs) of any grade were experienced by 139 patients (27.3%), categorized as follows: grade 1 by 96 patients (18.8%), grade 2 by 30 patients (5.9%), and grade 3 by 12 patients (2.4%). No grades 4–5 were observed. The most common cirAEs were rash eruptions in 69 patients (13.5), pruritus without visible rash in 37 patients (7.3%), and eczematous eruptions in 12 patients (2.4%). Regarding multisystem irAEs, 173 patients experienced multiple SOC irAEs, of which 110 patients included cirAE and 73 those that did not.

The incidence of cirAEs was higher in patients with better ECOG (34.5% in PS 0–1 vs. 23.6% in PS 2 and 14.2% in PS 3; *p* 0.018); also, when ICIs agents were combined with chemotherapy and in those treated in the first-line setting (30.6% in first-line, 25% in second line, and 13.4% in third-line treatment; *p* = 0.019). However, there were no significant differences for the rest of the clinicopathological characteristics between the cirAE and non-cirAE groups (Table 1).

### 3.3. Clinical Outcomes and cirAEs

Among patients evaluable for efficacy, as illustrated in Table 2, the objective response rate (ORR) was significantly higher in those who developed cirAEs. Of the 139 patients with cirAEs, 75 (54.3%) achieved a response to ICIs, while 9 (7.9%) experienced disease progression. An additional 52 patients (37.4%) achieved disease stabilization, resulting in a disease control rate (DCR) of 91.7%. In contrast, among 344 evaluable patients without cirAEs, only 101 (29.9%) responded to treatment, while 96 (28.1%) experienced disease progression (*p* = 0.0001). Furthermore, there was a significant association between ORR and OS in both groups, with and without cirAEs. However, the benefit was even greater in the last group, with more than 13 months of difference: See Table 2 and Appendix A, Table A1.

**Figure 1:**
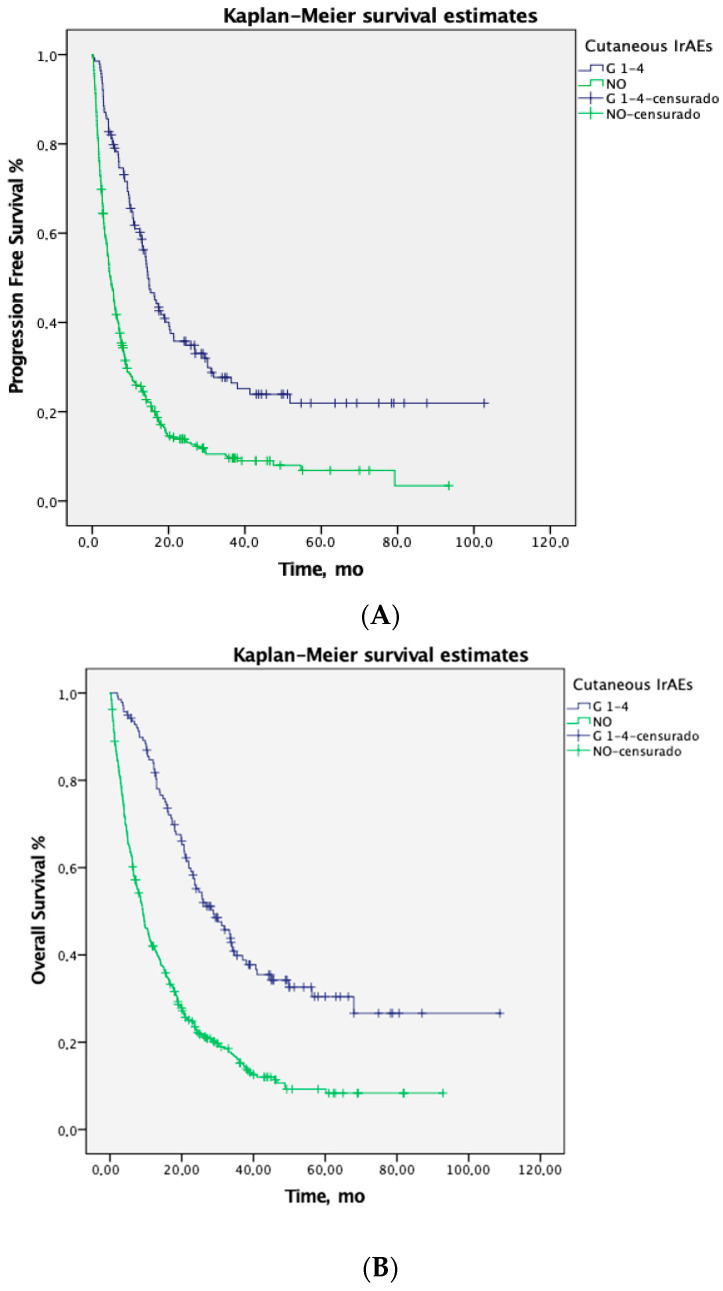
Kaplan-Meier survival estimates of progression-free (PFS) (**A**) and overall (OS) (**B**) survival for patients who experienced cirAE vs. those that did not. (**A**) Median PFS in patients with cirAE 14.6 m (IC95% 12.3-16.8) vs. 4.7 m (IC95% 3.9-5.5) in those that did not.; *p* = 0.0001); (**B**) Median OS in patients with cirAE 29 m (IC95% 21.3–33.6) vs. 9.2 m (IC95% 7.7–10.6; Log Rank (Mantel-Cox) (*p* = 0.0001).

At a median follow-up of 48 months, patients with cirAEs demonstrated significantly longer PFS and OS compared to those without cirAEs (Figure 1). The median PFS was 14.6 months (95% CI 12.3–16.8) in patients with cirAEs versus 4.7 months (95% CI 3.9–5.5) in those without cirAEs (*p* = 0.0001). The median OS in patients with cirAE was 29 months (95% CI 21.3–33.6) versus 9.2 months (95% CI 7.7–10.6) in those that did not; (*p* = 0.0001). Also, PFS and OS were significantly prolonged among patients experiencing cirAE grade 1 to 2 (median, PFS 14.9 months and OS 30 months) compared to patients with grade 3 (median, PFS 8.3 and OS 15.6 months) and those without cirAEs (median, PFS 4.8 and OS 9.1 months): See Table 3. Notably, the OS benefit associated with cirAEs was consistently observed across almost all subgroups (Table 4).

**Figure 2:**
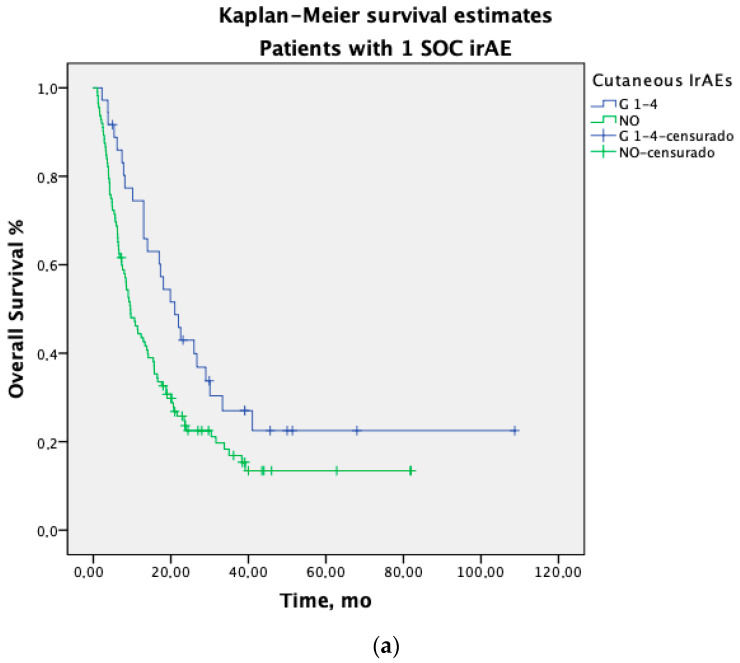

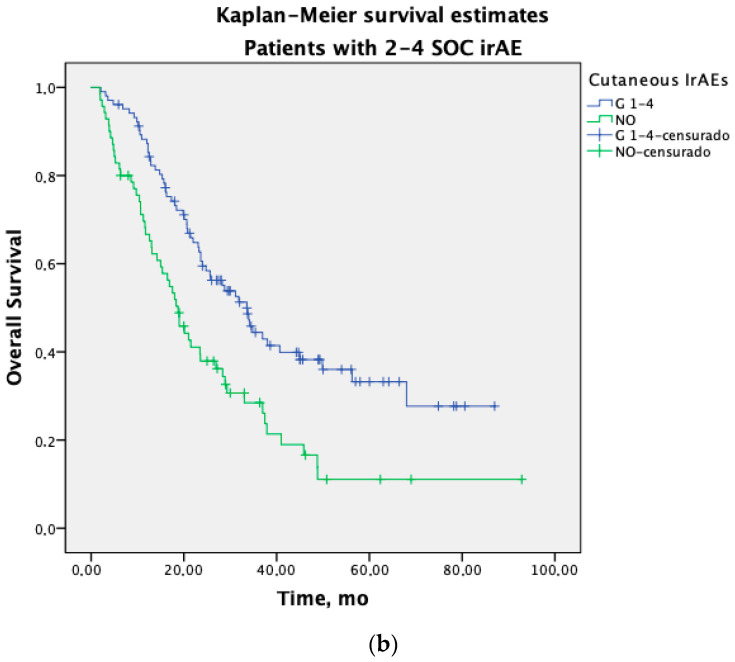
Kaplan–Meier overall survival (OS) estimates according to the number of system/organ classes (SOCs) affected (**a**) OS in patients with 1 SOC irAE who experienced cirAE, median OS 21 months (95% CI 14.9–27.1) vs. other irAEs, median 9.6 months (95% CI 7.1–12.1); *p* 0.023. HR: 0.60 (95% CI 0.39–0.93), *p*. 0.024 (**b**) OS in patients with ≥2 SOC irAEs who experienced cirAE, median OS 33.5 months (95% CI 27.3–39.8) vs. other irAEs, median 18.7 months (95% CI 15.1–22.2); *p* 0.001. HR: 0.51 (95% CI 0.35–0.74), *p*. 0.001.

**Figure A1:**
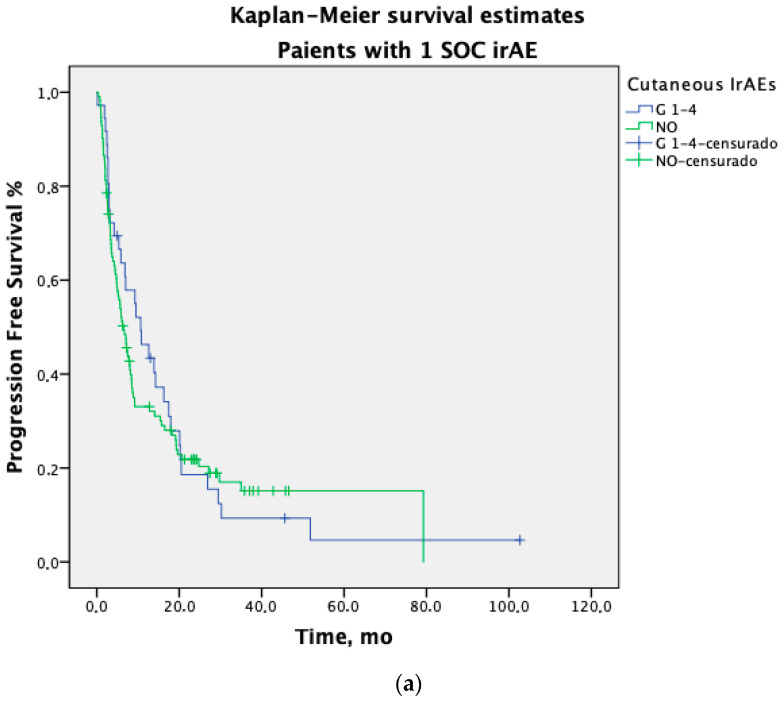

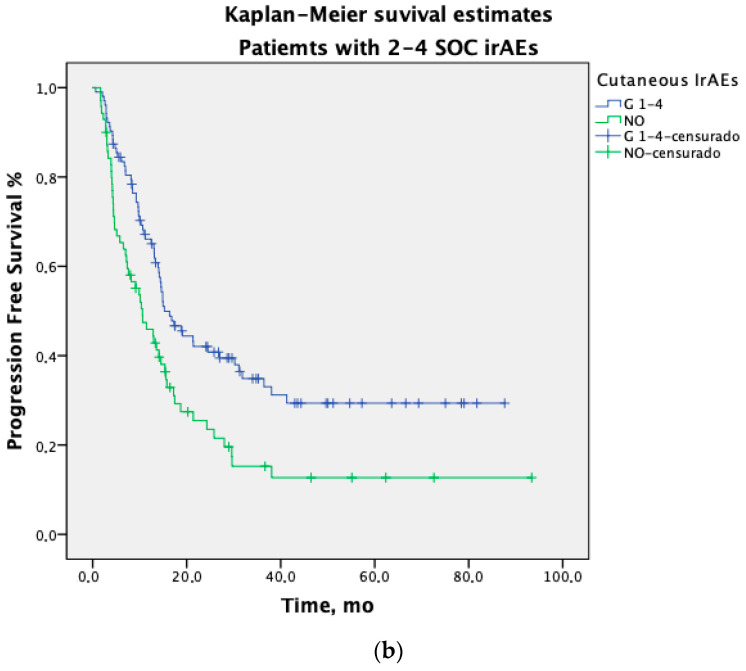
Kaplan–Meier progression-free survival (PFS) estimates, according to the number of system/organ class (SOC) affected (**a**) Median PFS in patients with 1SOC cirAE was 10.6 months (95% IC 4.2–17.1) vs. other SOC irAEs, median 6.5 months (95% CI 4.8–8.1); *p* = 0.637. HR: 1.11 (95% CI 0.96–1.29), *p* = 0.637; (**b**) Median PFS in patients with ≥2 SOC irAEs who experienced cirAE was 15.2 months (95% CI 11.1–19.4) vs. other irAEs, median 10.5 months (95% CI 6.4–14.7); *p* 0.002. PFS HR: 0.79 (95% CI 0.65–0.96), *p* = 0.016.

Additionally, to further examine the prognostic independence of the number of SOCs and cutaneous irAEs, we show a stratified analysis of PFS (Figure A1) and OS (Figure 2). The median PFS was significative longer in patients with ≥2 SOC irAEs who experienced cirAE than in those with other irAEs; 15.2 months (95% CI 11.1–19.4) vs. 10.5 months (95% CI 6.4–14.7); *p* = 0.002. PFS HR: 0.79 (95% CI 0.65–0.96), *p*. 0.016. The PFS in patients with only 1 SOC and cirAE was 10.6 months (95% CI 4.2–17.1) vs. 6.5 months (95% CI 4.8–8.1) with other SOCs; *p* = 0.637. HR:1.11 (95% CI 0.96–1.29), *p* = 0.637.

Patients who experienced multisystem irAEs involving cirAEs and other SOCs demonstrated progressively greater survival benefits. The median OS in patients with 1 SOC irAE was 9.6 months (95% CI 7.1–12.1), increasing to 21 months (95% CI 14.9–27.1) in patients who experienced cirAE; HR: 0.60 (95% CI 0.39–0.93), *p* = 0.024. This benefit is even greater in patients with more than two SOCs; the median OS was 18.7 months (95% CI 15.1–22.2), increasing to 33.5 months (95% CI 27.3–39.8) in patients who experienced cirAE; HR:0.51 (95% CI 0.35–0.74), *p* = 0.001.

### 3.4. Multivariable Analysis

We evaluated the relevance of cirAEs and multisystem SOCs with potentially influencing baseline parameters in a multivariate Cox regression analysis (Table 5). The presence of any cirAE was independently predicted/significantly associated with improved PFS (HR 0.74; 95% CI: 0.56–0.99, *p* = 0.044) and OS (HR 0.60; 95% CI: 0.44-0.80, *p* = 0.0001). Also, the presence of multisystem irAEs with more than two SOCs contained independent prognostic information for favorable/further enhanced PFS (HR 0.34; 95% CI: 0.25–0.46, *p* = 0.001 and OS (HRs 0.45; 95% CI: 0.34–0.61, *p* = 0.0001).

## 4. Discussion

This real-world study provides a comprehensive evaluation of the association between cirAEs and clinical outcomes in patients with advanced NSCLC treated with anti-PD-1/PD-L1 therapies. Our findings demonstrate that the development of cirAEs is significantly associated with prolonged OS, PFS, and higher ORR compared to patients without cirAEs.

Patients who developed cirAEs had a median OS of 29 months compared to 9.2 months in patients without cirAEs, and a median PFS of 14.6 months versus 4.7 months, respectively. Interestingly, multivariable analysis confirmed that the development of any cirAEs was independently associated with improved OS HR 0.60 (95% CI 0.44–0.80); *p* = 0.0001 and PFS HR 0.74, (95% CI 0.56–0.99); *p* = 0.044. These results are consistent with prior studies. For instance, a recent meta-analysis of 23 studies, comprising 22,749 patients treated with ICIs [22], reported a robust association between cirAE prevalence and improved survival outcomes across cancer types. A subgroup analysis of seven studies involving NSCLC also demonstrated significant improvements in OS (HR 0.50; 95% CI, 0.33–0.77; *p* = 0.002) and PFS (HR 0.61; 95% CI, 0.46–0.80; *p* < 0.001). In a second meta-analysis focusing on lung cancer, Wang D. et al. [14] also found that patients who experienced dermatological irAEs had longer OS: HR: 0.53, 95% CI (0.42–0.65), *p* < 0.00001 and PFS: HR: 0.51, 95% CI (0.43–0.61), *p* < 0.00001 than those who did not.

Despite the current reliance on the PD-L1 tumor proportion score (TPS) for selecting immunotherapy in NSCLC [7], no universally validated biomarkers exist for routine clinical use to predict treatment outcomes reliably [6,23]. Identifying biomarkers remains a critical challenge in clinical oncology and translational research. Our study suggests that cirAEs could serve as a novel clinical biomarker, providing oncologists with a practical tool to monitor treatment efficacy.

Overall, the cirAE rate of 27% was consistent with reported rates from previous studies [14,15,16]. The most common cirAEs were rash, pruritus, and eczematous eruptions, in line with a meta-analysis that reported a rate of 12.4% for rash and 10.4% for pruritus in NSCLC [24]. The occurrence of cirAE may reflect a robust systemic immune activation capable of overcoming the immunosuppressive tumor microenvironment. T-cell activation, which plays a dual role in mediating anti-tumor effects and immune-related toxicities, is central to this process.

In our cohort, patients with PS0 treated with ICIs in the first-line setting and those combing ICI with chemotherapy had an increased risk of developing cirAEs. Consistently with published data [16,17], the majority of cirAEs were grade 1–2, with only 2.4% of patients developing grade 3 toxicity. In some cases, cirAEs resolve spontaneously without requiring treatment discontinuation, highlighting the importance of careful monitoring. No life-threatening cirAEs were observed, and none of the patients with grade 3 cirAEs required hospitalization. Notably, patients with grade 1–3 cirAEs were significantly more likely to respond to ICI therapy compared to patients without cirAEs. These findings reinforce the notion that mild cirAEs, such as rash or pruritus, could serve as visible markers of effective anti-tumor immune responses.

Interestingly, we found that cirAE patients with higher numbers of affected systems had a notably better prognosis. In a study of 559 NSCLC patients receiving anti-PD1 immunotherapy, patients experiencing multiple-site irAEs had significantly longer PFS than those with single-site irAEs; however, OS was no longer increased in the multivariate analysis [25]. Another multicenter cohort study of 623 NSCLC patients treated with ICI demonstrates an association between multisystem irAEs and survival, although it is striking that patients with only one irAE did not experience a significant increase in OS compared to the non-irAE group [26]. Here, patients experiencing multisystem irAEs involving more than one SOC demonstrated progressively greater survival benefits in PFS 0.34 (95% 0.25–0.46) and OS 0.45 (0.34–0.61) compared to those with a single SOC or no irAEs. We hypothesize that the predictive role of multisystem irAEs might depend mainly on skin toxicity. Additionally, patients who experienced multisystem irAEs involving cirAEs vs. other SOC irAEs demonstrated progressively greater survival benefits in PFS HR: 0.79 (IC 95% 0.65–0.96), *p*. 0.016 and OS HR: 0.51(IC 95% 0.35–0.74), *p*. 0.001. These findings suggest that the development of multisystem cirAEs may reflect heightened immune activation and enhanced anti-tumor activity.

This retrospective observational study has several limitations. Imbalances between subgroups could not be avoided; the small numbers of patients with cirAE G3 make it difficult to reach robust conclusions between higher- and lower-grade skin toxicity. Third, our analysis demonstrates correlations rather than causal results. In order to reduce the heterogeneity, we conducted Cox regression adjusted by relevant baseline characteristics. To our knowledge, these are the first data to describe the promising association of cirAEs and several SOC irAEs with ICI efficacy in advanced NSCLC. Based on our findings, we could also speculate that recognizing cirAEs as indicators of a favorable response may reshape treatment strategies, advocating for a more proactive approach to diagnose and treat other SOC irAEs early in a reversible phase. Allowing mild toxicities to persist under close monitoring may help sustain treatment benefits without compromising quality of life rather than discontinuing therapy prematurely.

## 5. Conclusions

In conclusion, this study confirms that cirAEs may serve as a useful marker for predicting the efficacy of ICIs in NSCLC patients. The development of multisystem cirAEs strongly correlates with a greater survival benefit, indicating that these events could help identify patients most likely to benefit from ICI therapy.

## Figures and Tables

**Table 1:** Baseline patient characteristics.

Characteristics	All Patients *n* = 510	cirAE *n* = 139	Non-cirAE *n* = 371	*p*-Value
Age				0.288
<65	263 (51.5)	75 (53.9)	188 (50.7)	
>65	247 (48.5)	64 (45.1)	183 (49.3)	
Gender				0.186
Male	390 (76.5)	102 (73.4)	288 (77.6)	
Female	120 (23.5)	37 (26.6)	83 (22.4)	
Smoker				0.502
Former/Never smoker	282 (55.3)	77 (55.4)	205 (55.3)	
Current smoker	228 (44.7)	62 (44.6)	166 (44.7)	
Histology				0.743
Adenocarcinoma	311 (61)	87 (62.6)	224 (60.4)	
Squamous	175 (34.3)	47 (33.8)	128 (34.5)	
Undifferentiated Cell	24 (4.7)	5 (3.6)	19 (5.1)	
PdL1%				0.290
>50	102 (20)	29 (20.8)	73 (19.7)	
1–49	111 (21.8)	37 (26.6)	74 (19.9)	
<1	194 (38.1)	45 (32.4)	149 (40.2)	
Unknown	75 (14.7)	28 (20.1)	75 (20.2)	
Kras				0.130
Wt	159 (31.2)	44 (31.6)	115 (31)	
mutated	55 (10.8)	21 (15.1)	34 (9.2)	
Unknown	296 (58)	74 (53.2)	222 (59.8)	
ECOG performance status				0.018
0	171 (33.5)	59 (42.4)	112 (30.2)	
1	317 (62.2)	76 (54.7)	241 (65)	
2	21 (4.1)	3 (2.1)	18 (4.8)	
Number of therapies				0.028
First line	310 (60.7)	95 (68.3)	215 (57.9)	
Second line	148 (29.1)	37 (26.6)	111 (30)	
Third line	52 (10.2)	7 (5.1)	45 (12.1)	
Type of treatment				0.037
ICI	320 (62.7)	78 (56.1)	242 (65.2)	
ICI + Chemotherapy	190 (37.3)	61 (43.9)	129 (34.8)	
Number of SOC irAEs				0.0001
1 SOC	148 (46.2)	36 (25.8)	112 (61.5)	
2–4 SOC	173 (53.8)	103 (74.2)	70 (38.5)	

Data are presented as *n* (%), unless otherwise specified. Non-cirAE, non-cutaneous immune-related adverse event; PD-L1, programmed death ligand-1; Wt, Will type; ECOG, Eastern Cooperative Oncology Group; ICI, immune checkpoint inhibitors; SOC, system/organ classes.

**Table 2:** Best responses to ICI treatment.

Recist Criteria	All Patients	cirAE/Non-cirAE	*p*-Value	cirAE OS (95% CI)	*p*-Value	Non-cirAE OS 95% CI	*p*-Value
Partial Response	176 (34.5)	75/101	0.001	36.9 (12.8–61.1)	0.0001	23.7 (14.8–32.6)	0.0001
Stable disease	193 (37.8)	54/133		20.8 (16.7–24.8)		10.4 (8.6–12.1)	
Progression disease	105 (20.6)	9/96		7.8 (6.6–9.0)		4.1 (3.3–4.9)	
Not evaluable	36 (7.1)	1/35		2.2 (0.7–1.3)		1.1 (0.6–1.4)	

Data are *n* (%; 95% CI) or *n* (%), unless stated otherwise. CI, confidence interval; cirAE, cutaneous immune-related adverse event; ICI, immune checkpoint inhibitors.

**Table 3:** Univariate analysis of prognostic factors for progression-free survival and overall survival.

Characteristics	All Patients *n* 510 (100%)	PFS Median (95% CI)	*p*-Value	OS Median (95% CI)	*p*-Value
Age			0.267		0.061
<65	263 (51.5)	7.06 (5.7–8.3)		15.3 (12.0–18.7)	
>65	247 (48.5)	5.9 (4.5–7.4)		11.2 (8.4–14.1)	
Gender			0.560		0.046
Male	390 (76.5)	6.7 (5.4–8.1)		12.8 (1.4)	
Female	120 (23.5)	6.9 (5.6–8.2)		16.6 (2.3)	
Smoking status			0.063		0.042
Former/Never smoker	282 (55.3)	5.8 (4.1–7.4)		12.2 (9.3–15.1)	
Current smoker	224 (43.9)	7.3 (5.9–8.7)		16 (12.3–19.6)	
Histology			0.036		0.033
Adenocarcinoma	311 (61)	7.6 (6.4–8.7)		15.3 (12.1–18.6)	
Squamous	175 (34.3)	5.6 (4.6–6.6)		12.2 (9.6–14.4)	
Undifferentiated Cell	24 (4.7)	4.4 (0.1–11.1)		12.8 (0.1–27.1)	
PdL1%			0.016		0.047
>50	102 (20)	8.1 (4.1–12.2)		15 (11.5–18.4)	
1–49	111 (21.8)	8.9 (7.8–9.9)		18 (11.3–24.6)	
<1	194 (38.1)	6.5 (5.3–7.6)		13.8 (7.9–19.6)	
Unknown	103 (20.1)	4.2 (2.1–6.3)		9.1 (7.1–11.1)	
Kras			0.001		0.001
Wt	159 (31.2)	8.6 (6.1–11.2)		17.9 (14.5–21.2)	
mutated	55 (10.8)	10.3 (0.7–19.9)		31.6 (16.9–46.3)	
Unknown	296 (58)	5.0 (4.0–6.1)		10.4 (8.4–12.3)	
ECOG performance status			0.110		0.003
0	171 (33.5)	7.9 (5.7–10.2)		18.8 (15.2–22.3)	
1	317 (62.2)	6.5 (5.4–7.5)		12.2 (10.2–14.2)	
2	22 (4.3)	4.6 (0.1–10.7)		7.3 (0.1–14.5)	
Number of therapies			0.001		0.0001
First line	310 (60.7)	9.1 (7.6–10.6)		17.3 (14.6–20.0)	
Second line	148 (29.1)	4.2 (2.4–6.1)		9.5 (7.2–11.8)	
Third line	52 (10.2)	2.9 (2.6–3.2)		6.8 (2.4–11.2)	
Type of treatment			0.0001		0.005
ICI	320 (62.7)	4.7 (3.7–5.7)		10.5 (7.8–13.1)	
ICI + Chemotherapy	190 (37.3)	10.6 (7.5–3.6)		17.3 (14.0–20.6)	
Cutaneous irAE			0.0001		0.0001
cirAE	139 (51.5)	14.6 (12.3–16.8)		29 (21.3–33.6)	
None	371 (48.5)	4.7 (3.9–5.5)		9.2 (7.7–10.6)	
Cutaneous irAE			0.0001		0.0001
G1	96 (18.8)	14.9 (10.3–19.3)		29 (22.0–35.9)	
G2	30 (5.9)	14.9 (10.2–19.6)		31.1 (10.9–51.2)	
G3	12 (2.3)	8.3 (1.1–15.5)		15.6 (7.6–10.6)	
G4	-	-		-	
Non-cirAE	372 (73)	4.8 (4.0–5.6)		9.1 (7.6–10.6)	
Number of SOCs			0.0001		0.0001
1	173 (33.9)	7.1 (5.4–8.7)		13 (8.7–17.2)	
2–4	148 (29.0)	14.4 (12.7–16.1)		25.7 (19.2–32.1)	
None	189 (37.1)	2.83 (2.3–3.3)		6.2 (4.5–7.9)	

CI, confidence interval; cirAE, cutaneous immune-related adverse event; ECOG, Eastern Cooperative Oncology Group; PD-L1, programmed death ligand-1.

**Table 4:** Forest plot for subgroup analysis of the association between cutaneous immune-related adverse event (cirAE) development and overall survival.

Subgroup	Sample	HR (95% CI)	*p*-Value
Overall	510 (100)	0.40 (0.31–0.51)	0.0001
Age < 65	263 (51.5)	0.69 (0.57–0.85)	0.0001
Age > 65	247 (48.5)	0.77 (0.64–0.93)	0.0002
Male	390 (76.5)	0.77 (0.66–0.88)	0.0001
Female	120 (23.5)	0.63 (0.45–0.87)	0.001
Former/Never smoker	282 (55.3)	0.76 (0.64–0.90)	0.0001
Current smoker	224 (43.9)	0.69 (0.55–0.87)	0.0001
Adenocarcinoma	311 (61)	0.71 (0.59–0.87)	0.0001
Squamous	175 (34.3)	0.72 (0.58–0.89)	0.0001
Undifferentiated Cell	24 (4.7)	1.09 (0.65–1.81)	0.634
PdL1 > 50	102 (20)	0.67 (0.49–0.92)	0.002
PdL1 1–49	111 (21.8)	0.64 (0.46–0.90)	0.004
PdL1 < 1	194 (38.1)	0.70 (0.53–0.91)	0.002
Unknown	75 (14.7)	0.93 (0.79–1.10)	0.454
Kras Wt	159 (31.2)	0.63 (0.46–0.86)	0.001
Kras mutated	55 (10.8)	0.52 (0.29–0.93)	0.012
ECOG 0	171 (33.5)	0.73 (0.58–0.93)	0.003
ECOG 1	317 (62.2)	0.72 (0.60–0.87)	0.0001
ECOG 2	21 (4.1)	1.12 (0.95–1.32)	0.729
First line	310 (60.7)	0.74 (0.62–0.89)	0.000
Second line	148 (29.1)	0.71 (0.55–0.92)	0.001
Third line	52 (10.2)	0.89 (0.66–1.22)	0.358
ICI	320 (62.7)	0.73 (0.61–0.86)	0.011
ICI + Chemotherapy	190 (37.3)	0.75 (0.59–0.96)	0.0001
1 SOC irAE	320 (62.7)	0.73 (0.61–0.86)	0.011
≥2 SOC irAEs	190 (37.3)	0.75 (0.59–0.95)	0.0001
Partial Response	176 (34.5)	0.77 (0.59–1.02)	0.067
Stable disease	193 (37.8)	0.79 (0.66–0.95)	0.003
Progression disease	105 (20.6)	0.99 (0.81–1.21)	0.646

HR, hazard ratios; CI, confidence interval. Forest plot for subgroup analyses of overall survival. HR < 1 favors the cirAE group.

**Table 5:** Multivariable Cox proportional hazard model analysis for PFS and OS with relevant baseline characteristics and cirAE.

Baseline Characteristics	PFS HR (95% CI)	*p*-Value	OS HR (95% CI)	*p*-Value
Age				0.469
<65 vs. >65 years	-		0.92 (0.75–1.14)	
Gender				0.580
Female vs. Male	-		0.93 (0.72–1.19)	
Smoker		0.092		0.039
Current vs. Former/Never	0.84 (0.68–1.02)		0.80 (0.64–0.98)	
Histology		0.014		0.057 0.698 0.018
Adenocarcinoma	0.85 (0.51–1.4)	0.53	0.89 (0.51–1.55)	
Squamous	0.72 (0.58–0.9)	0.03	0.75 (0.60–0.95)	
Undifferentiated Cell	ref		ref	
PdL1%		0.746		0.900
>50	0.88 (0.64–1.20)	0.429	0.92 (0.67–1.26)	0.611
1–49	0.85 (0.60–1.19)	0.356	0.89 (0.63–1.16)	0.539
<1	0.97 (0.67–1.13)	0.872	0.99 (0.69–1.42)	0.666
Unknown	ref		ref	
Kras		0.218 0.450 0.085		0.021 0.110 0.008
Wt	0.91 (0.71–1.16)		0.80 (0.62–1.05)	
mutated	0.72 (0.50–1.04)		0.57 (0.38–0.86)	
Unknown	ref		ref	
ECOG	-			0.005
0			0.43 (0.26–0.73)	0.002
1			0.53 (0.32–0.87)	0.013
2			ref	
Number of therapies		0.645 0.728 0.733		0.410 0.194 0.255
First line	0.92 (0.60–1.41)		0.76 (0.51–1.14)	
Second line	1.06 (0.73–1.56)		0.80 (0.55–1.16)	
Third line	ref		ref	
Type of treatment				
ICI + Chemotherapy vs. ICI	0.86 (0.64–0.96)	0.307	0.99 (0.73–1.33)	0.956
Cutaneous irAE		0.044		0.0001
G1–4 vs. Non	0.74 (0.56–0.99)		0.60 (0.44–0.80)	
Number of SOC irAEs		0.0001 0.0001 0.0001		0.0001 0.006 0.0001
1 SOC	0.51 (0.40–0.66)		0.69 (0.54–0.90)	
2–4 SOCs	0.34 (0.25–0.46)		0.45 (0.34–0.61)	
None	ref		ref	

HR, Hazard Ratios CI, confidence interval; PD-L1, programmed death ligand-1; Ref, reference; Wt, Will type; ECOG, Eastern Cooperative Oncology Group; cirAE cutaneous immune-related adverse event; ICI, Immune checkpoint inhibitors; SOC, system/organ classes.

## Data Availability

The original contributions presented in this study are included in the article. Further inquiries can be directed to the corresponding author(s).

## Abbreviations

The following abbreviations are used in this manuscript:

MDPI	Multidisciplinary Digital Publishing Institute
CTCAE	Common Terminology Criteria for Adverse Events
CTLA-4	Cytotoxic T-lymphocyte antigen-4
NSCLC	Non-small-cell cancer
cirAEs	Cutaneous immune-related adverse event
ECOG	Eastern Cooperative Oncology Group
PDL-1	Programmed death ligand-1
DOAJ	Directory of open access journals
PD-1	Programmed cell death protein-1
ICIs	Immune checkpoint inhibitors
ORR	Objective Response Rate
PFS	Progression-Free Survival
SOC	System/organ class
CR	Completed response
CI	Confidence interval
HR	Hazard Ratios
OS	Overall survival
PR	Partial response
LD	Linear dichroism
Wt	Will type

## Appendix A

**Table A1:** Survival analysis based on response rate.

Recist Criteria	All Patients *n* 510 (100%)	PFS Median +/− (IC 95%)	*p*-Value	OS Median +/− (IC 95%)	*p*-Value
Partial Response	176 (34.5)	19.6 (14.3–24.8)	0.0001	33.1 (27.638.4)	0.0001
Stable disease	193 (37.8)	7.0 (6.1–7.8)		13.3 (10.5–16.1)	
Progression disease	105 (20.6)	1.7 (1.4–2.1)		4.6 (3.6–5.5)	
Not evaluable	36 (7.1)	1.1 (0.7–1.3)		1.2 (0.9–1.5)	

Data are *n* (%; 95% CI) or *n* (%), unless stated otherwise. Percentages might not add up to 100% due to rounding. CI, confidence interval; cirAE, cutaneous immune-related adverse event; ICI, Immune checkpoint inhibitors.
